# Morbidity and doctor characteristics only partly explain the substantial healthcare expenditures of frequent attenders: a record linkage study between patient data and reimbursements data

**DOI:** 10.1186/1471-2296-14-138

**Published:** 2013-09-17

**Authors:** Frans T Smits, Henk J Brouwer, Aeilko H Zwinderman, Jacob Mohrs, Hugo M Smeets, Judith E Bosmans, Aart H Schene, Henk C Van Weert, Gerben ter Riet

**Affiliations:** 1 Department of General Practice - Academic Medical Center, University of Amsterdam, Amsterdam 1105 AZ, The Netherlands; 2 Department of Clinical Epidemiology, Biostatistics and Bioinformatics - Academic Medical Center, University of Amsterdam, Amsterdam 1100 DD, The Netherlands; 3 Julius Centre for Health Sciences and Primary Care, University Medical Center, Utrecht 3584 CG, The Netherlands; 4 Agis Health Database, Agis Zorgverzekeringen, Amersfoort 3800 HA, The Netherlands; 5 Department of Health Sciences and EMGO Institute for Health and Care Research, Faculty of Earth and Life Sciences, VU University Amsterdam, Amsterdam 1081 BT, The Netherlands; 6 Department of Psychiatry, Academic Medical Center, Amsterdam 1100 DD, The Netherlands

**Keywords:** (Persisting) frequent attender, High utilizer, Healthcare expenditure, Primary care, General practice, Primary care physician, General practitioner, Linkage study, Reimbursements data, Multimorbidity

## Abstract

**Background:**

Frequently attending patients to primary care (FA) are likely to cost more in primary care than their non-frequently attending counterparts. But how much is spent on specialist care of FAs? We describe the healthcare expenditures of frequently attending patients during 1, 2 or 3 years and test the hypothesis that additional costs can be explained by FAs’ combined morbidity and primary care physicians’ characteristics.

**Methods:**

Record linkage study. Pseudonymised clinical data from the medical records of 16 531 patients from 39 general practices were linked to healthcare insurer’s reimbursements data. Main outcome measures were all reimbursed primary and specialist healthcare costs between 2007 and 2009. Multilevel linear regression analysis was used to quantify the effects of the different durations of frequent attendance on three-year total healthcare expenditures in primary and specialist care, while adjusting for age, sex, morbidities and for primary care physicians characteristics. Primary care physicians’ characteristics were collected through administrative data and a questionnaire.

**Results:**

Unadjusted mean 3-year expenditures were 5044 and 15 824 Euros for non-FAs and three-year-FAs, respectively. After adjustment for all other included confounders, costs both in primary and specialist care remained substantially higher and increased with longer duration of frequent attendance. As compared to non-FAs, adjusted mean expenditures were 1723 and 5293 Euros higher for one-year and three-year FAs, respectively.

**Conclusions:**

FAs of primary care give rise to substantial costs not only in primary, but also in specialist care that cannot be explained by their multimorbidity. Primary care physicians’ working styles appear not to explain these excess costs. The mechanisms behind this excess expenditure remain to be elucidated.

## Background

Primary care physicians (PCP) spend a disproportionate amount of their time on a relatively small proportion of patients who frequently attend their practice [1,2]. In most studies these frequent attenders (FAs) are defined as the upper 10% of the most frequently consulting patients per sex and age group [3-5].

Of all FAs during one year, 15.4% continues to be a frequent attender during 3 years (1.6% of all enlisted patients) [2,6]. All FAs during one year (10% of all enlisted patients by definition) were responsible for 39% of all consultations while the 1.6% FAs during 3 consecutive years (persistent FAs; pFAs) were responsible for 8% of all consultations [2].

FAs, and pFAs in particular, usually have multiple (chronic) somatic diseases, psychiatric disorders and social problems [2,7,8]. FAs are more frequently referred to specialist care than non-frequent attenders (non-FAs) [9]. However, little is known about the magnitude of the differences in healthcare utilisation and costs between non-FAs and FAs and between subgroups of FAs in *specialist* care. Possibly these differences in healthcare costs can be explained by specific characteristics and morbidities of FAs, and by PCP characteristics. If not, detection and treatment of underlying, not yet detected, conditions in FAs may result in a better quality of life of FAs and decrease in costs.

The aim of this paper is to describe primary and specialist care costs of FAs in primary care using a combination of clinical and healthcare insurer’s data and to examine associations between healthcare expenditures of FAs of different duration in primary care and patient’s morbidities and PCP characteristics.

## Methods

### Design and data collection

In a historic three-year cohort study seven primary healthcare centres in Amsterdam, The Netherlands, provided data. These centres participate in the PCP-based continuous morbidity registration network of the Department of General Practice at the Academic Medical Centre of the University of Amsterdam (HAG-net-AMC).

Of all patients, 45% were insured by one health insurer: AGIS [10]. Only these data were used. Reimbursement claims of all insured people are electronically verified and saved at the patient level in the AGIS Health Database. This registration provides data of treatments by PCPs, specialists, other health professionals and prescriptions.

### Linking of both databases

The two databases were linked using a number that uniquely identifies single Dutch citizens, the so-called “burger [citizen] service nummer [number]” or BSN in Dutch. Through a certified trusted third party that specializes in record linkage through irreversible pseudonymisation (ZorgTTP, Houten, The Netherlands), the PCP database (clinical data) and the insurer database (financial reimbursement data) were encrypted. Next, both encrypted databases were linked. This resulted in a database in which individual patients could not be identified.

### Study population

All patients of 18 years and older registered at the participating PCPs in 2009 were eligible for this study. Patients were classified according to their frequent attendance status. FAs were defined as those patients whose attendance rate ranked in the top 10th centile of four age groups (18–30 years; 31–45 years; 46–60 years; 61 years+) for men and women separately [3]. Frequent attendance was determined for each of the years 2007, 2008 and 2009. FAs during one year (1yFAs) were classified as patients who attended frequently during one of those years, FAs during 2 years (2yFAs) as patients who attended frequently in two of these years and FAs during three years (3yFAs; pFAs) as those who attended frequently during all three years. Patients who were not a frequent attender in any of these years (non frequent attenders; non-FAs) were used as a reference group.

### Ethics approval

The study was conducted according to the Dutch legislation on data protection (Ministry of Justice, the Netherlands). Ethics approval was waived by the Medical Ethics Committee of the Academic Medical Center of the University of Amsterdam.

### Variables

#### Costs

In the Netherlands, health insurance covers a broad range of healthcare costs including PCP care, prescription medication, specialist care in and outside hospitals and emergency care. Only over the counter medication such as simple painkillers and antihistamines are excluded. EU citizens who work and live in the Netherlands usually have Dutch healthcare insurance and were included in our study if they were AGIS insured. All Dutch citizens are required by law to have a healthcare insurance. Total costs of all reimbursed primary and specialist care costs in the years 2007–2009 were used as the dependent variable. Costs were retrieved from the insurer’s database and covered all care reimbursed to their clients during these years. By taking the sum of all three years we tried to account for fluctuating costs because of temporary diseases.

We divided primary care costs in somatic costs (e.g. PCP) and psychological costs (psychologists). Costs of specialist care consist of all specialist remuneration (in and outside hospitals) and costs of hospital admissions. Mental health prescriptions have an Anatomical Therapeutic Chemical (ATC) Classification System code and are included as mental health costs, both in primary and specialist care. Costs of homecare, district nurses and nursing homes were not included.

#### Attendance

Patients were classified based on the number of face-to-face consultations with the PCP. Because we wanted to study consultation behaviour consultations with other practice staff were excluded because these contacts are almost always initiated by the PCP or his staff and concern planned control of chronic diseases.

#### Morbidity

The presence of morbidity was assessed using PCPs’ registration of medical problems. Any medical problem which needed long-term medical attention or monitoring lasting or likely to last for more than 6 months and/or which recurred more than 4 times per half year was added to the PCPs' electronic medical record and coded according to the ICPC system [11,12]. These EMR data were extracted for the purpose of this study. The validity and reliability of coding of the problem list in our PCP network has been shown to be good in previous studies [4,5]. The problem lists were extracted at the end of 2009.

We selected a subset of conditions that according to the literature are associated with frequent attendance and may also be associated with costs (confounding): diabetes mellitus, cardiovascular disease, respiratory disease, cancers, locomotor problems, skin problems and digestive problems (feelings of) anxiety, (feelings of) depression, addictions, other psychological/psychiatric problems, all social problems and medically unexplained symptoms (MUS) [2,4,13,14]. MUS were defined according to Robbins et al. and covered several locomotor problems [15]. See Additional file 1.

### PCPs’ characteristics and practice style

PCPs’ characteristics and practice style were measured using a questionnaire (Additional file 2), the mean number of registered medical problems and consultations per listed patient (adjusted for age and sex differences between the practices) and the PCP practice size (4 categories: <1000 patients; 1001–1250 patients; 1251–1500 patients; >1500 patients).

### Ethnicity

Ethnicity of the most prevalent groups in Amsterdam (Dutch, Surinamese, Ghanese, Morrocan and Turkish) was determined in the insurer’s database using automated name recognition algorithms manually checked by employees of ethnic descent.

### Statistical analysis

We compared primary and specialist care costs between 1yFAs, 2yFAs, pFAs and non-FAs. Associations between costs and patient characteristics were estimated using multilevel linear regression analysis with PCP as random intercept. Differences between patient groups for categorical variables were analysed using generalized linear mixed models. Statistical significance was set at P < 0.05. SPSS 20.0 for windows was used for the statistical analysis.

A linear model for the actual costs implied that we modelled the mean costs. This facilitated the easy interpretation of the regression parameters, namely the cost difference associated with one unit change of the characteristic. Mean costs are also relevant for policy-makers and health insurance management because of the close relationship between average and total costs. Because the cost-distribution is highly skewed, we provided the median costs as the statistic that is better suited for individual patients. We also considered several transformations to normalize the costs distributions using the Box-Cox family of transformations, and found that a power close to zero yielded the best transformation (that is, the logarithmic transformation) both for the primary and the specialist care costs [16-18]. The distributions of the transformed costs were however not much better than those of the untransformed costs. The results of the regression analyses were qualitatively similar for transformed and untransformed costs. Multivariate analysis was applied to determine independent predictors of costs. To determine whether PCP characteristics were associated with costs, we extended the mixed-effects models with PCP characteristics.

## Results

### Linkage

Of the eligible PCP patients 2% could not be linked to the insurers’ database by missing numbers in the files of the PCPs or administrative failures.

### Frequent attenders

In 2009 data were available on 16 531 patients. Of these patients 1208 were not enlisted in 2008 and/or 2007. Of all 16 531 patients in 2009, 2540 were classified as 1yFAs, 843 as 2yFAs, and 334 as 3yFAs, and 12 814 were non-FAs. Characteristics and medical complaints are summarized in Table 1. FAs were older and more often female than non-FAs. A non-Dutch ethnic background was slightly more prevalent among FAs. The number of all medical complaints, both somatic and psychological, was higher among FAs (in particular among pFAs) than among non-FAs.

**Table 1:** **Description of the study population in 2009** (**by frequent attender**-**status**)

	**Non**-**FA**^**1**^	**1yFA**^**1**^	**2yFA**^**1**^	**3yFA**^**1**^
Number of patients	12 814	2540	843	334
Mean age	46 (18) ^2^	47 (18)	49 (19)	53 (17)
Females, n(%)	7371 (58)	1410 (56)	508 (60)	215 (64)
Ethnicity				
Dutch, n(%)	9798 (77)	1872 (74)	603 (72)	235 (70)
Moroccan, n(%)	465 (4)	120 (5)	49 (6)	17 (6)
Turkish, n(%)	280 (2)	66 (3)	24 (3)	12 (4)
Surinamese, n(%)	2262 (18)	480 (19)	167 (20)	70 (21)
Mean number of entries on the problem list in 2009 (SD)^3^				
All problems	1.58 (2.02)	2.43 (2.54)	3.39 (2.82)	4.50 (3.34)
social	0.02 (0.16)	0.04 (0.20)	0.06 (0.26)	0.07 (0.26)
psychological	0.14 (0.40)	0.24 (0.50)	0.35 (0.61)	0.56 (0.76)
depression	0.03 (0.18)	0.06 (0.23)	0.10 (0.30)	0.13 (0.34)
anxiety	0.02 (0.14)	0.03 (0.18)	0.05 (0.23)	0.10 (0.32)
Medically unexplained symptoms	0.09 (0.33)	0.17 (0.45)	0.26 (0.54)	0.36 (0.66)
Diabetes mellitus	0.09 (0.29)	0.14 (0.35)	0.18 (0.39)	0.19 (0.40)
Respiratory diseases	0.13 (0.36)	0.16 (0.40)	0.25 (0.47)	0.30 (0.51)
Cardiovascular diseases	0.27 (0.63)	0.42 (0.74)	0.59 (0.92)	0.66 (0.88)

^1^Different frequent attender groups: Patients who never frequently attended during 3 years (non-FAs); patients who attended frequently during 1 year (1yFA), 2 years (2yFA), or 3 years (3yFA).

^2^Numbers in brackets are standard deviations, unless indicated otherwise.

^3^Mean number of entries per patient on the problem list. Patient could have more than one (social, cardiovascular, etc.) problem. SD means Standard Deviation.

### Healthcare costs

Median and mean costs of both primary and specialist care were significantly and substantially higher in all FA groups than in non-FAs (p < 0.001). Summed over the three years the mean primary care costs of 1yFAs, 2yFAs, 3yFAs and non-FAs were 2650, 3872, 4674 and 1645 Euros respectively. Specialist care costs showed a similar pattern: 5866, 6911, 11 150 and 3399 Euros, respectively (all differences p < 0.001). Costs that could be attributed to psychosocial complaints were much lower than costs attributed to somatic complaints in all groups but showed a similar trend over the four patient groups (p < 0.001). See Table 2.

**Table 2:** **Median and mean 3**-**year costs in Euros per patient in primary and specialist care** (**by frequent attender status**)^#^

	**Frequent attender status**							
	**Non**		**1 year**		**2 years**		**3 years**	
**Characteristics**	**Median**	**Mean (SD)**	**Median**	**Mean (SD)**		**Mean (SD)**	**Median**	**Mean (SD)**
Primary care physician (PCP)	283	338 (197)	444	525 (300)	613	722 (402)	810	1005 (664)
Emergency care by the PCP	-	20 (164)	0	51 (114)	0	83 (156)	63	156 (334)
Physical therapy^1^	0	41 (379)	0	77 (558)	0	99 (624)	0	181 (931)
Complementary medicine^1^	0	16 (98)	0	24 (122)	0	37 (142)	0	43 (160)
Laboratory costs	0	19 (49)	0	42 (69)	40	65 (89)	52	86 (101)
Medication:			0		0		0	
psychotropic medication	0	86 (719)	0	134 (760)	0	156 (670)	16	233 (672)
antibiotics	0	15 (182)	11	28 (72)	19	40 (79)	33	63 (108)
painkillers	0	19 (165)	0	52 (358)	0	50 (305)	16	99 (296)
other medication	135	1091 (4019)	401	1717 (4724)	756	2621 (6745)	1262	2808 (4542)
Somatic primary care costs	511	1553 (4212)	1049	2501 (5052)	1696	3696 (7035)	2626	4421 (5316)
Psychological primary care costs	0	92 (721)	0	48 (764)	0	176 (677)	20	253 (679)
All primary care costs	540	1645 (4284)	1137	2650 (5145)	1839	3872 (7056)	2746	4674 (5583)
All specialist care costs	379	3399 (14124)	1753	5866 (16752)	3023	6911 (12877)	5393	11 150 (18 582)

^#^All differences between means (and medians) were statistically significant at the <0.001 level.

^1^Only the costs reimbursed by the insurer.

### Patient-related determinants of healthcare costs

Univariate associations between patient characteristics and healthcare costs and multivariate adjustment are summarized in Additional file 3 and Additional file 4. Differences between PCPs were negligible. The intraclass correlation (PCP-variance as part of the total variance) was smaller than 0.001 for both primary and specialist care.

After multivariate adjustment for all patient and PCP characteristics large and significant cost differences remained between the different FA categories not only in primary care, but even more in specialist care with extra expenditures for pFAs of 1268 and 4025 Euros respectively (see Table 3).

**Table 3:** **The effects of frequent attender status on mean costs in primary and specialist care adjusted for all patient characteristics and morbidities (in Euros) additional costs**^**#**^

	**Primary care difference (SE)**	**Specialist care difference (SE)**
Non-Frequently Attending Patients (reference)^a^	0	0
1-year Frequent Attenders	481 (44)	1242 (117)
2-year Frequent Attenders	800 (73)	1897 (192)
3-year Frequent Attenders	1268 (115)	4025 (302)

^#^All effects were statistically significant at the <0.001 level.

Adjusted for sex, ethnicity, age, number of active problems, diabetes, respiratory, cardiovascular, social, psychological and medically unexplained problems, cancer, locomotor, skin and digestive problems.

^a^Mean costs for non-FAs:1645 Euros (all primary care) and 3399 Euros (all specialist care).

### PCP-related determinants of healthcare costs

Thirty-nine PCPs of seven primary healthcare centres participated in this study. The average practice size was 1312 patients (range, 312–2714) and 82% of PCPs participated in medical education or research.

After correction for patient characteristics, intraclass correlations between healthcare costs of individual patients in the same general practice were small: 0.011 for primary care costs, 0.006 for secondary care costs. See Table 4.

**Table 4:** **Effect of primary care physicians**’ **characteristics on 3**-**year mean costs** (**in Euros**) **of primary and specialist healthcare**

		**Primary care difference in costs (SE)**	**Specialist care difference in costs (SE)**
Male sex of PCP^¶^, n(%)	15 (38) ^a^	−10 (20) ^a^	−100 (66) ^a^
Involvement in education of medical students and/or vocational PCP training, n(%)	32 (82)	−5 (36)	59 (91)
General characteristics ^b^			
Practice size^c^	2.4 (1.1)	−23 (9)	−35 (32)
Experience (years)^d^	17 (9)	−1 (1.1)	−0.2 (4)
Mean number of active problems per patient on problem list	1.7 (0.5)	−73 (17)	−289 (51)
Mean number of contacts (adjusted for age and sex of the patient)	2.8 (0.3)	13 (39)	−80 (131)
Mean percentage of all problems that was psychological or social	12 (3)	−1 (93)	2 (11)
Special interest in managing^e^			
Diabetes mellitus	3.2 (0.7)	0 (16)	24 (52)
COPD ^f^/Asthma	3.1 (0.7)	−23 (14)	−50 (45)
Cardiovascular disease	3.0 (0.5)	−20 (18)	−146 (58)
Anxiety	2.9 (0.8)	−11 (13)	−80 (41)
Depression	3.0 (0.7)	−15 (14)	−68 (46)
Medically Unexplained Symptoms	4.0 (0.7)	10 (14)	33 (47)

^¶^PCP indicates primary care physician.

^a^Numbers in brackets are standard errors, unless indicated otherwise.

^b^In Euros per unit of the scale of the characteristic.

^c^Divided in 4 classes: class 1:0–1000 patients; class 2:1001–1250 patients; class 3:1251–1500 patients; class 4:>1500 patients. Range 312–2,714 patients.

^d^Per (additional) year experience.

^e^Five levels of interest (from 1 (no special interest) to 5 (very much interest)) and 5 levels of percentage of patients treated by the GP (0%-100%). See Additional file 2.

^f^Chronic Obstructive Pulmonary Disease.

## Discussion

### Summary

In this population of 16 531 Dutch primary care patients costs for FAs, and in particular pFAs, were considerably higher than for non-FAs throughout the healthcare. After multivariable correction for thirteen demographic and medical confounding factors at the patient and physician level, frequent attendance remained associated with higher expenditures both in primary and specialist care.

### Strength of this study

As far as we know this study is the first to combine clinical primary care data and PCP characteristics with cost data in both primary and specialist care in this particular type of patients. Second, as most PCPs participated in the registration network for over 15 years and received regular feedback on their registration, we think these data are of good quality, especially for somatic and psychiatric (DSM-IV axis 1) problems [11]. However, registration of e.g. personality disorders is expected to be less complete. Third, the cost data were collected from an insurance company and are a valid reflection of the healthcare costs of the selected patients [10]. The population covered by this healthcare insurer represents the urbanized area very well [10]. Fourth, we used a proven method of encrypting of both databases performed by an independent third party. Of all patients only 2% could not be linked. Some of these patients may have been illegal and not insured.

The distributions of patient characteristics of key interest and of the confounders guaranteed ample analytical contrast [19]. For example, age varied between 18 and 101, 58% were female, and 20 percent were of Surinam origin. This resulted in enough power to robustly estimate the effect of these factors on costs.

### Limitations of this study

Registration and coding of medical problems in primary care has limitations. In general, within a General Practice Research Network, one can distinguish several factors to explain morbidity and prescription differences: “healthcare system”, “methodological characteristics of the network”, “general practitioner”, and the “patient”. These factors and sub-factors are often interrelated and explain the different prevalence figures [20,21]. Because PCPs register medical problems mostly during consultations, the number of registered problems could be underreported in non-FAs (few contacts) or overreported (if resolved problems are not removed) in FAs (many contacts). Overreporting may also occur in recurrent or temporary diseases and may lead to overestimation of prevalence and underestimation of the effect on costs. As in our registry, the problem lists are subject to regular evaluation, we think that the errors caused by this are likely to be small. Second, the variation in PCPs’ characteristics was modest and this reduced the data-analytic contrast at the PCP level. For example, eighty-two percent of the PCPs were involved in education or research and all practices were relatively small (mean 1,312 patients; average Dutch practice: 2,150 patients). In addition, PCPs might have tended to answer in a socially desirable way to the questions on their special interests. This may have led to a seemingly homogeneous group of PCPs and to underestimation of the effects of special interests on costs. Third, there may be undocumented determinants of costs (residual confounding). By incorporating an extensive set of possible confounders in the analysis we tried to diminish this bias. However, we had no data on severity of diseases, perceived health status, quality of life, illness attitude, life events and socio-economic level. The resulting residual confounding may have led to overestimation of the association of (p)FA-ship and costs. Fourth, because 7% of the patients selected in 2009 were not enlisted all 3 years we may have slightly underestimated the number and the effect of pFAs. Fifth, because insurers compete on the basis of their premium, the patient’s choice of the AGIS health insurer could cause a some degree of self-selection. Finally, this study originates in the specific Dutch healthcare system with a well-organized primary care in which PCPs provide continuity of care and act as gatekeepers to specialist care. This may restrict the generalisability of our results to countries with a similar healthcare system like the United Kingdom [8].

### Comparison with existing literature

As most researchers we chose a proportional definition of frequent attendance that takes into account age and sex [3-5]. Earlier research has shown that the number of health problems is consistently and positively associated with utilisation of primary and specialist care [8,22]. Our study confirms these findings and evaluates the influence of clinical and physician characteristics on the relationship between frequent attender status and healthcare costs.

The mechanism behind the interaction between FAs and PCP prompting the performance of additional diagnostic and therapeutic actions is not fully understood. Earlier theories emphasized a negative interaction between some patients and their PCP in maintaining “somatic fixation” resulting in unnecessary consultations, tests and treatments [23-25]. In patients with MUS explanations for ‘somatisation’ should be sought in doctor-patient interaction rather than in patients’ psychopathology [26-28]. However, the adjusted differences in costs between the PCPs participating in this study were small. This suggests that patient determinants may be more important in explaining extra expenditures by FAs than PCP characteristics, although a larger and more diverse group of PCPs may be needed to corroborate this. The nestor of Dutch general practice, Frans Huygen, already demonstrated that families tend to be consistent in illness and consultations patterns [29-31]. This may imply that frequent attending also could be understood as a kind of learned behaviour or trait.

### Implications for research and/or practice

This study shows that FAs of the PCP are also heavy users of all clinical services. As most somatic problems in this patient group are already dealt with in chronic care models, most advantages are likely to be gained by diagnosing and treating undetected psychiatric and social problems. Adequate diagnosis and treatment of such problems in primary care and optimal PCP-patient-communication may prevent referral of patients to specialist care and may strengthen the gate keeping role of PCPs [32-36]. The assumed trait character of frequent attendance may impede the effects of any treatment.

Future research should focus on the aetiology of (the persistence of) frequent attendance [36,37]. Personality factors, life events, social support and socio-economic level should be investigated more to assess how they affect attendance and costs. Moreover, we need to clarify how and why patients prompt their physicians to do more than strictly indicated. Armed with more knowledge about these causes of frequent attendance future randomised trials may target those interventions aimed at modifying these causes and reduce illness, attendance and costs.

## Conclusions

Frequent attenders of primary care contribute substantially to costs not only in primary care but also in specialist care. Morbidity, social problems and PCP characteristics appear to only partly explain these expenditures. Frequent attendance may therefore be considered as an independent, yet incompletely understood contributing determinant of healthcare utilisation and costs in primary and specialist care.

## Abbreviations

FAs: Frequent attenders; 1yFAs: Frequent attenders during 1 year; 2yFAs: Frequent attenders during 2 years; 3yFAs: Frequent attenders during 3 years; pFAs: Persistent frequent attenders (=3yFAs); PCP: Primary care physician; COPD: Chronic obstructive pulmonary disease; ICPC: International classification of primary care; MUS: Medically unexplained symptoms.

## Competing interest

The authors declare that they have no competing interests. All authors declare: no support from any organisation for the submitted work; no financial relationships with any organisation that might have an interest in the submitted work in the previous years, no other relationships or activities that could appear to have influenced the submitted work.

## Authors’ contributions

FTS drafted and designed the article, monitored data collection, analysed the data, and drafted and revised the paper. He is guarantor. GtR designed the article, analysed the data and drafted en revised the paper. He is guarantor. HJB monitored the patient data collection, analysed these data and read and revised the paper. AHZ wrote the statistical analysis plan, analysed the data, and drafted and revised the paper. JM collected and monitored the patient data collection, analysed these data and read and revised the paper. HMS monitored the healthcare costs data collection, analysed these data and read and revisited the paper. JEB helped to design our analytic methods of costs analysis, analysed the data and read and revised the paper. AHS and HCW supervised our project, helped to design the study, read and revised the paper. All authors read and approved the final version of this paper.

## Authors’ information

FTS works as a PCP in Health Center Reigersbos, Amsterdam, The Netherlands since 1982. In 2009 he started a PhD at the department of General Practice of the university of Amsterdam, The Netherlands on Frequent Attenders (PERFACTIO project).

## Pre-publication history

The pre-publication history for this paper can be accessed here:

http://www.biomedcentral.com/1471-2296/14/138/prepub

## Supplementary Material

Additional file 1 Selected problems and diseases with ICPC-code. Click here for file

Additional file 2 Primary care physicians’ questionnaire. Click here for file

Additional file 3 Univariate associations between patient characteristics and 3-year costs in primary and specialist care. Click here for file

Additional file 4 The multivariable effects of patient characteristics on mean costs (by healthcare level). Click here for file
