# Supplementary material for: Morbidity and doctor characteristics only partly explain the substantial healthcare expenditures of frequent attenders: a record linkage study between patient data and reimbursements data
Source: BMC Fam Pract. 2013 Sep 17;14:138. doi: 10.1186/1471-2296-14-138 (PMC3851974; doi:10.1186/1471-2296-14-138)
Supplement: Additional file 2 — Primary care physicians’ questionnaire. [file 1471-2296-14-138-S2.doc]

**Additional file 2:** **Primary care physicians’ questionnaire**

The interaction between patient and physician factors may determine the number of visits of a patient. With this questionnaire we are trying to determine primary care physicians’ characteristics which may influence patients’ attendance.

Filling out this survey will take about 10 minutes of your time. Please answer all questions. After entering, your data will, of course, be anonymized.

Thank you very much for your effort!

1. In which year did you graduate from medical school?

2. At what university did you graduate as a doctor?

3. Please note the number of years you work as a physician: …

4. I am currently working

- part-time

- full-time

5. If part-time, what percentage of a full contract do you work?: …

6. Do you have special knowledge of/interest in a specific part of general practice?

If so, in which specific field are you interested? (Several options are possible)

() paediatric medicine

() geriatric medicine

() management of the practice

() financial management

() professional organizations

7. Do you have special knowledge of/interest in (the treatment of) diabetes mellitus?

( ) no special interest

( ) little interest

( ) normal interest

( ) more than normal interest

( ) very much interest

8. Do you have special knowledge of/interest in (the treatment of) asthma and/or COPD?

( ) no special interest

( ) little interest

( ) normal interest

( ) more than normal interest

( ) very much interest

9. Do you have special knowledge of/interest in (the treatment of) cardiovascular diseases?

( ) no special interest

( ) little interest

( ) normal interest

( ) more than normal interest

( ) very much interest

10. Are you a member of a guideline committee of the Dutch College of General Practitioners (NHG)?
 () Yes
 () No

11. If "Yes": Please give the title of the guideline(s)
 NHG-standard: ________________
 NHG-standard: ________________
 NHG-standard: ________________


12. Are you involved in the organization of continuous medical education/post academic training of primary care physicians?
 () Yes
 () No


13. Do you treat patients with anxiety disorders?

Please indicate what percentage of patients with anxiety disorders you manage yourself.
 () 0%
 () 25%
 () 50%
 () 75%
 () 100%

14. Do you treat patients with depressive symptoms or depression?

Please indicate what percentage of patients with depressive symptoms you manage yourself.
 () 0%
 () 25%
 () 50%
 () 75%
 () 100%


15. Do you treat patients with medically unexplained somatic complaints? (Think for instance of unexplained pain)

Please indicate what percentage of patients with unexplained symptoms you manage yourself.
 () 0%
 () 25%
 () 50%
 () 75%
 () 100%


16. Are you a primary care physician vocational trainer?
 () Yes
 () No


17. Do you regularly give (vocational) training to medical students ?
 () Yes
 () No


18. Are you also working at a university and/or the Dutch College of General Practitioners (NHG)? Multiple options are possible.

() Yes, university
 () Yes, NHG
 () No

19. Do you cooperate with other primary care physicians (PCPs)?
 () Group practice (only PCPs)
 () Health centre
 () PCPs with one electronic medical file
 () I am a solo PCP


20. Do you work with a trained practice nurse?
 () Yes
 () No


21. If so, how many days support these practice nurses your practice?

____________________________________________

22. Do you work with another discipline?

Please check the appropriate checkbox (multiple options).
 () Nurse
 () Social psychiatric nurse
 () General social worker
 () Primary care psychologist
 () Other: …..


23. How do you organize your office schedule?

Check on the checkboxes (multiple options).
 () Only after appointment
 () An open schedule
 () Telephone consultation
 () E-mail consultation
 () appointments via the Internet


24. My age is:

____________________________________________

25. I am:
 () Man
 () Woman


Thank you very much for completing this questionnaire!
