# Supplementary material for: Morbidity and doctor characteristics only partly explain the substantial healthcare expenditures of frequent attenders: a record linkage study between patient data and reimbursements data
Source: BMC Fam Pract. 2013 Sep 17;14:138. doi: 10.1186/1471-2296-14-138 (PMC3851974; doi:10.1186/1471-2296-14-138)
Supplement: Additional file 3 — Univariate associations between patient characteristics and 3-year costs in primary and specialist care. [file 1471-2296-14-138-S3.doc]

**Additional file 3:** **Univariate associations between patient characteristics and 3-year costs in primary and specialist care**

|  |  | Primary care | | Specialist care | |
| --- | --- | --- | --- | --- | --- |
|  | Mean (range) | difference (SE) | P-value | difference (SE) | P-value |
| Age ^a^ | 47 (18-98) | 59 (2) | < 0.001 | 101 (6) | < 0.001 |
| Male (reference) |  | 0 |  | 0 |  |
| Female |  | 343 (74) | < 0.001 | 155 (231) | 0.50 |
| Ethnicity: |  |  |  |  |  |
| Dutch (reference) |  | 0 |  | 0 |  |
| Moroccan |  | -409 (186) | 0.001 | -1212 (590) | 0.17 |
| Turkish |  | -369 (244) | 0.001 | -709 (764) | 0.17 |
| Surinamese |  | -318 (96) | 0.001 | -190 (300) | 0.17 |
| Problems on the problem list^b^ |  |  |  |  |  |
| Number of problems | 2.43 (0-18) | 743 (15) | < 0.001 | 1466 (50) | < 0.001 |
| Diabetes | 0.14 (0-1) | 3146 (116) | < 0.001 | 4003 (369) | < 0.001 |
| COPD/Asthma | 0.16 (0-2) | 1132 (96) | < 0.001 | 929 (302) | 0.002 |
| Cardiovascular | 0.42 (0-5) | 1684 (52) | < 0.001 | 4210 (165) | < 0.001 |
| Social | 0.04 (0-2) | 244 (212) | 0.25 | 660 (649) | 0.32 |
| Psychological | 0.24 (0-3) | 1183 (81) | < 0.001 | 4506 (254) | < 0.001 |
| Depression | 0.06 (0-1) | 1292 (179) | < 0.001 | 2602 (559) | < 0.001 |
| Anxiety | 0.03 (0-1) | 886 (231) | < 0.001 | 1423 (723) | 0.049 |
| Addiction | 0.05 (0-2) | 1235 (186) | < 0.001 | 5617 (582) | < 0.001 |
| Other psychological | 0.10 (0-2) | 1404 (128) | < 0.001 | 6711 (400) | < 0.001 |
| Medically Unexplained Symptoms | 0.17 (0-5) | 947 (96) | < 0.001 | 1791 (300) | < 0.001 |
| Frequent attender during: |  |  |  |  |  |
| Non-FAs (reference) |  | 0 |  | 0 |  |
| 1 year |  | 1005 (109) | <0.001 | 2467 (355) | <0.001 |
| 2 years |  | 2227 (246) | <0.001 | 3512 (461) | <0.001 |
| 3 years |  | 3029 (308) | <0.001 | 7751 (1024) | <0.001 |

_________________________________________________________________________________________________

^a^ Costs per additional year of age

^b^ Binary variables were modeled using a dummy; all other variables were modelled linearly
