# Supplementary material for: Morbidity and doctor characteristics only partly explain the substantial healthcare expenditures of frequent attenders: a record linkage study between patient data and reimbursements data
Source: BMC Fam Pract. 2013 Sep 17;14:138. doi: 10.1186/1471-2296-14-138 (PMC3851974; doi:10.1186/1471-2296-14-138)
Supplement: Additional file 4 — The multivariable effects of patient characteristics on mean costs (by healthcare level). [file 1471-2296-14-138-S4.doc]

**Additional file 4. The multivariate effects of patient characteristics on mean costs (by healthcare level^#^)**

|  |  | | Primary Care | | | Specialist Care | |
| --- | --- | --- | --- | --- | --- | --- | --- |
|  | Mean (range) ^4^ | difference (SE) | | P-value | difference (SE) | | P-value |
|  |  |  | |  |  | |  |
| Age^1^ | 47 (18-98) | 8.1 (1.1) | | < 0.001 | -4.5 (3.0) | | 0.13 |
| Sex |  |  | |  |  | |  |
| Male (reference) |  | 0 | |  | 0 | |  |
| Female |  | 119 (33) | | < 0.001 | 259 (86) | | 0.002 |
| Ethnicity |  |  | |  |  | |  |
| Dutch (reference) |  | 0 | |  | 0 | |  |
| Moroccan |  | 171 (131) | | 0.087 | 7 (343) | | 0.54 |
| Turkish |  | -41 (11) | |  | 194 (290) | |  |
| Surinamese |  | -36 (106) | |  | 38 (277) | |  |
| Problems on the problem list^¶^ | | | | | |  | |
| Total Number | 2.43 (0-18) | 451 (17) | | < 0.001 | 802 (44) | | < 0.001 |
| Diabetes mellitus | 0.14 (0-1) | 1330 (59) | | < 0.001 | -268 (155) | | 0.083 |
| COPD^2^/Asthma | 0.16 (0-2) | 161 (46) | | 0.001 | -660 (121) | | < 0.001 |
| Cardiovascular | 0.42 (0-5) | 263 (36) | | < 0.001 | 659 (94) | | < 0.001 |
| Social | 0.04 (0-2) | -512 (95) | | < 0.001 | -582 (249) | | 0.019 |
| Psychological | 0.24 (0-3) | 365 (282) | | 0.196 | 1074 (740) | | 0.15 |
| Depression | 0.06 (0-1) | -401 (292) | | 0.17 | -433 (767) | | 0.57 |
| Anxiety | 0.03 (0-1) | -472 (263) | | 0.072 | -588 (689) | | 0.39 |
| Addiction | 0.05 (0-2) | -344 (295) | | 0.24 | -329 (775) | | 0.67 |
| Other psychological | 0.10 (0-2) | 191 (289) | | 0.51 | 428 (758) | | 0.57 |
| Medically Unexplained Symptoms | 0.17 (0-5) | -279 (50) | | < 0.001 | -801 (131) | | < 0.001 |
| Cancer | 0.04 (0-1) | 635 (82) | | < 0.001 | 4195 (217) | | < 0.001 |
| Locomotor | 0.16 (0-1) | -81 (57) | | 0.15 | 290 (148) | | 0.05 |
| Skin | 0.09 (0-1) | -289 (61) | | < 0.001 | -1124(159) | | < 0.001 |
| Digestive | 0.11 (0-1) | 113 (60) | | 0.059 | 492 (156) | | 0.002 |
|  |  |  | |  |  | |  |
| Intraclass correlation (PCP level ^3^) |  | 0.0097 | |  | 0.0041 | |  |

______________________________________________________________________________________________________________________________

^#^ Based on the same regression analysis as presented in Table 4

^¶^ All variables were linear, unless indicated otherwise

^1^ Costs in Euros per unit increase (for instance per extra year of age)

^2^ chronic obstructive pulmonary disease

^3^ Variance between primary care physicians (PCPs) as part of the total variance (residual variance + PCP variance).

^4^ Frequent attenders during 1 year in 2009 .
